# D-4F increases microRNA-124a and reduces neuroinflammation in diabetic stroke rats

**DOI:** 10.18632/oncotarget.20751

**Published:** 2017-09-08

**Authors:** Ruizhuo Ning, Poornima Venkat, Michael Chopp, Alex Zacharek, Tao Yan, Xu Cui, Don Seyfried, Jieli Chen

**Affiliations:** ^1^ Department of Neurology, Henry Ford Hospital, Detroit, MI, USA; ^2^ Department of Neurology, First Hospital Harbin, Harbin, China; ^3^ Department of Physics, Oakland University, Rochester, MI, USA; ^4^ Gerontology Institute, Neurology, Tianjin Medical University General Hospital, Tianjin Neurological Institute, Key Laboratory of Post-Neurotrauma Neurorepair and Regeneration in Central Nervous System, Ministry of Education and Tianjin City, Tianjin, China

**Keywords:** stroke, diabetes mellitus, apolipoprotein-A1, microRNA-124a, neuroinflammation

## Abstract

D-4F is an apolipoprotein-A1 mimetic peptide that promotes anti-inflammatory effects. MicroRNA-124 is the most abundant brain-specific microRNA and has anti-inflammatory effects. In this study, we investigated the therapeutic efficacy and mechanisms of D-4F treatment of stroke in type one diabetes mellitus (T1DM) rats. Male Wistar rats were induced with T1DM, subjected to embolic middle cerebral artery occlusion and treated with PBS or D-4F (1 mg/kg i.p.) at 2, 24 and 48 hours after stroke (n=8/group). A battery of function tests, brain blood barrier (BBB) integrity, white matter changes and microRNA expression were evaluated *in vivo* and *in vitro*. D-4F treatment in T1DM-stroke rats significantly improves functional outcome, decreases BBB leakage, increases tight junction protein expression, decreases white matter damage and inflammatory factor expression, while increasing anti-inflammatory M2 macrophage polarization in the ischemic brain. D-4F significantly increases microRNA-124a expression, and decreases matrix metalloproteinase-9, tumor necrosis factor-α and toll-like receptor-4 gene expression in the ischemic brain, and in primary cortical neuronal and microglial cultures. Inhibition of microRNA-124 in cultured primary cortical neurons and microglia attenuates D-4F induced anti-inflammatory effects and M2 macrophage polarization. D-4F treatment of T1DM-stroke increases microRNA-124 expression, promotes anti-inflammatory effects and M2 macrophage polarization, which may contribute to D-4F-induced improvement in neurological function, and BBB and white matter integrity.

## INTRODUCTION

Diabetes mellitus (DM) patients suffer from a 3-4 fold higher risk of stroke and a worse vascular prognosis compared to non-DM patients [1, 2]. In DM patients, treatment of stroke with tPA (tissue plasminogen activator) increases the risk of death and spontaneous intra-cerebral hemorrhage [3]. Compared to non-DM stroke rodents, DM-stroke rodents suffer increased inflammatory effects in the ischemic brain, exacerbated proinflammatory responses, and increased release of cytotoxic enzymes, which together increase BBB leakage and brain hemorrhage [3, 4]. DM rats treated with tPA suffer from increased lesion volume, blood brain barrier (BBB) leakage, brain hemorrhagic transformation as well as worse functional outcome [3, 5–7]. Therefore, it is important to develop and test therapeutic approaches to specifically reduce neurological deficits after stroke in the DM population.

MicroRNAs (miR) are short sequences of non-coding RNA that can regulate several genes, pathways, and biological networks by acting in concert with other miRs or as stand-alone mediators [8]. In the pathogenesis of stroke, miRs are emerging as mediators of neurodegeneration and neuroinflammation [8, 9]. Among the known miRs, miR-124a is the most abundant brain specific miR, and miR-124a promotes neuronal differentiation during central nervous system (CNS) development [10]. MiR-124a is highly expressed by resident microglia in the CNS, mediates microglial quiescence in the CNS, and has anti-inflammatory effects [11, 12]. In patients with stroke, a significant decrease in serum miR-124 expression was found within 24 hours post stroke, and decreased miR-124 correlated with increased infarct volume and neuroinflammatory factor matrix metalloproteinase 9 (MMP9) expression [13]. MiR-124 up-regulation also decreases other neuroinflammatory factors such as tumor necrosis factor-α (TNFα) [14] and toll-like receptors (TLR) [15]. Additionally, miR-124 promotes macrophage polarization by decreasing the proinflammatory M1 phenotype and increasing the anti-inflammatory M2 phenotype [16].

In the pathogenesis of DM, and in DM-stroke complications, dysfunction of high-density lipoprotein (HDL) cholesterol increases inflammation [17], and the activated inflammatory responses induce extensive vascular damage [18]. Many of HDL's atheroprotective functions are due to Apolipoprotein A-1 (ApoA-1) [19]. ApoA-1 is widely studied as a mediator of cholesterol efflux and for its use as an anti-inflammatory agent. In a rat DM model, oral administration of D-4F, an ApoA-I mimetic peptide, enhances the functionality of the HDL particle without increasing serum HDL-C levels [20], promotes the conversion of HDL from a proinflammatory to an anti-inflammatory state [21, 22], and improves vascular reactivity [23]. To date, there are no investigations whether D-4F treatment in the DM-stroke population can promote functional outcome, and on the mechanisms of D-4F induced beneficial effects. In this study, we investigate whether D-4F treatment of stroke promotes miR-124a expression, and modulates neuroinflammatory effects and improves functional outcome after stroke in type one DM (T1DM) rats.

## RESULTS

### Stroke treatment using D-4F in T1DM rats does not decrease lesion volume and brain hemorrhagic transformation, but significantly decreases BBB leakage and improves functional outcome

**Figure 1:**
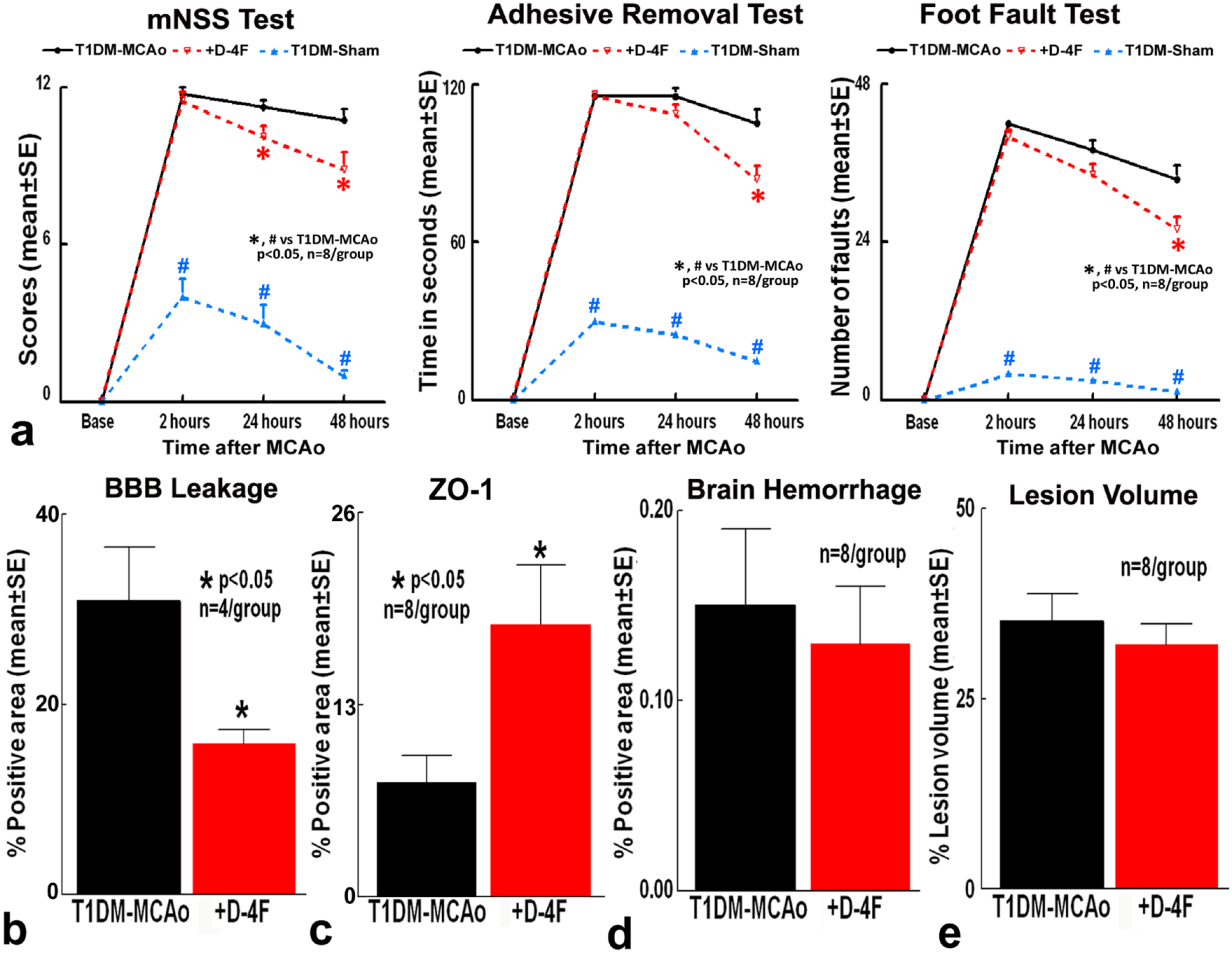
Stroke treatment using D-4F in T1DM rats does not decrease lesion volume and brain hemorrhagic transformation, but significantly decreases BBB leakage and improves functional outcome Stroke in T1DM rats induces significant neurological deficits compared to T1DM-sham control rats (^#^p<0.05; n=8/group). D-4F treatment in T1DM stroke rats significantly: **(a)** improves neurological functional outcome, **(b)** decreases BBB leakage, and **(c)** increases tight junction protein Zona-occluden-1 (ZO-1) expression around blood vessels, without significantly affecting **(d)** brain hemorrhage and **(e)** lesion volume compared to PBS treated T1DM stroke rats (^*^p<0.05, n=8/group) at 48 hours after stroke. Data are represented as mean ± SE.

To test the effects of D-4F treatment in T1DM-stroke rats, a battery of functional tests were performed. The modified neurological severity score (mNSS) test is a composite test that evaluates motor, sensory, balance and reflex actions. The foot-fault test evaluates sensorimotor function, motor coordination and limb placement deficits during locomotion; while the adhesive removal test evaluates somatosensory deficits. Stroke in T1DM rats significantly induces neurological deficits compared to T1DM rats subject to sham surgery (n=8/group, ^#^p<0.05, Figure 1a). D-4F treatment in T1DM stroke rats significantly decreases modified neurological severity score at 24 and 48 hours after stroke, improves somatosensory function in the adhesive removal test, and decreases the number of foot-faults in the foot-fault test at 48 hours after stroke (n=8/group, ^*^p<0.05, Figure 1a) compared to PBS treated T1DM rats.

To investigate whether D-4F treatment induced neurological functional improvement may be derived from decreased ischemic burden, hemorrhage or BBB protection, the infarct volume, BBB permeability and hemorrhagic transformation were measured. D-4F treatment in T1DM-stroke rats significantly decreases BBB leakage (n=4/group, ^*^p<0.05, Figure 1b) and significantly increases tight junction protein ZO-1 (Zone occluden-1) expression around blood vessels in the ischemic border zone at 48 hours after stroke compared to phosphate buffered saline (PBS) treated T1DM stroke rats (n=8/group, ^*^p<0.05, Figure 1c). BBB leakage was not detected in T1DM-sham control rats. D-4F treatment in T1DM-stroke rats does not decrease brain hemorrhagic transformation (n=8/group, Figure 1d) and lesion volume (n=8/group, Figure 1e) compared to PBS treated T1DM stroke control rats at 48 hours after stroke.

### D-4F treatment of stroke in T1DM decreases white matter damage

**Figure 2:**
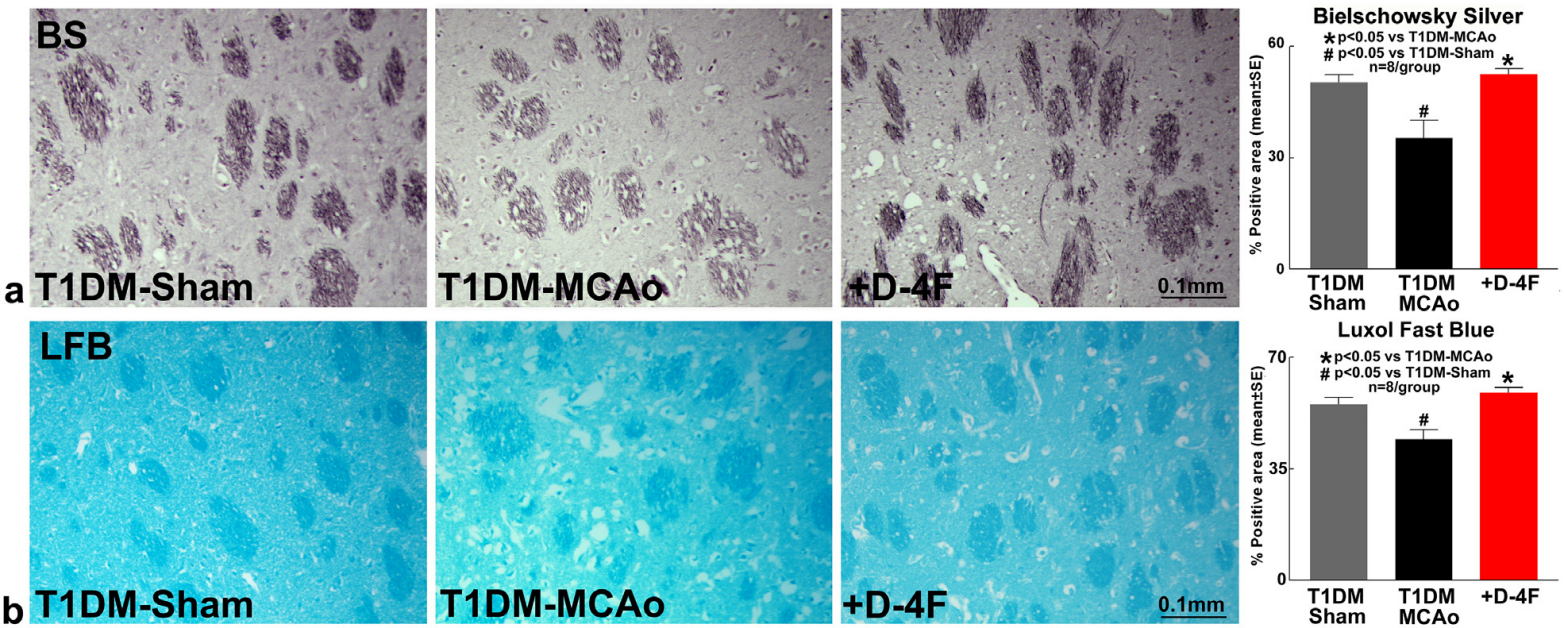
D-4F treatment of stroke in T1DM rats decreases white matter damage Stroke in T1DM rats significantly decreases axon (Bielschowsky silver staining) and myelin density (Luxol fast blue staining) compared to T1DM-sham control rats (^#^p<0.05; n=8/group). D-4F treatment of stroke in T1DM rats significantly increases **(a)** axon density (Bielschowsky silver staining) and **(b)** myelin density (Luxol fast blue staining) in the ischemic border zone compared to PBS treated T1DM stroke control rats (^*^p<0.05, n=8/group) at 48 hours after stroke. Data are represented as mean ± SE.

To test whether D-4F treatment promotes white matter (WM) remodeling, Bielschowsky silver (BS) and Luxol fast blue (LFB) staining were performed. Stroke in T1DM rats significantly decreases axon (BS, Figure 2a, ^#^p<0.05, n=8/group) and myelin (LFB, Figure 2b, ^#^p<0.05, n=8/group) density compared to T1DM-sham control rats. D-4F treatment of stroke in T1DM rats significantly increases axon (BS, Figure 2a, n=8/group, ^*^p<0.05) and myelin (LFB, Figure 2b, n=8/group, ^*^p<0.05) density in the ischemic border zone (IBZ) compared to PBS treated T1DM stroke rats at 48 hours after stroke.

### D-4F treatment of stroke in T1DM rats significantly decreases inflammatory factor expression and promotes M2 macrophage polarization in the ischemic brain

**Figure 3:**
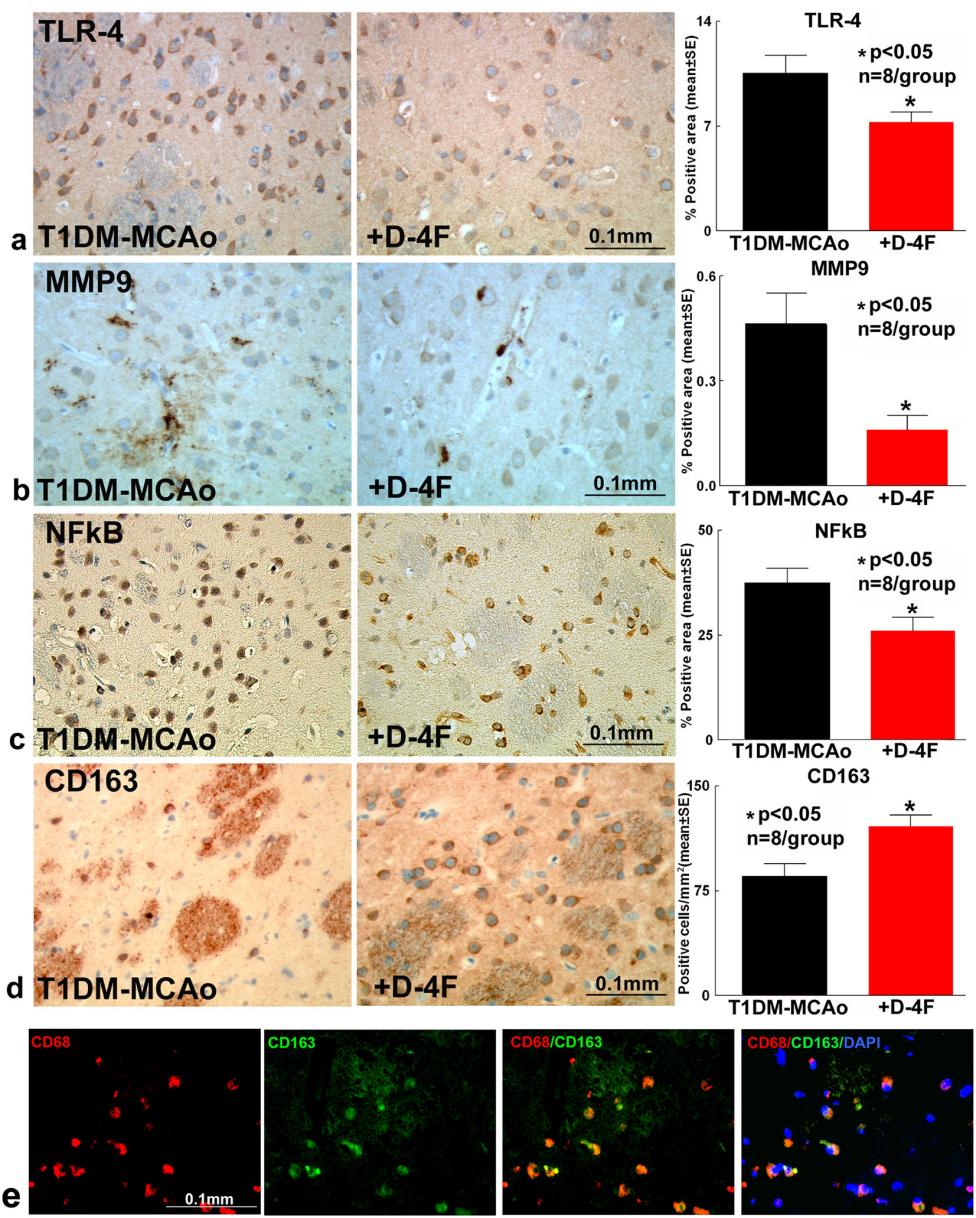
D-4F treatment of stroke in T1DM rats significantly decreases inflammatory factor expression and promotes M2 macrophage polarization in the ischemic brain At 48 hours after stroke, D-4F treatment significantly decreases **(a)** TLR4, **(b)** MMP9 and **(c)** NFκB expression compared to PBS treated T1DM stroke control rats (^*^p<0.05, n=8/group). **(d)** D-4F treatment also significantly promotes M2 macrophage polarization which is identified by increased CD163 expression compared to PBS treated T1DM stroke control rats (^*^p<0.05, n=8/group). Data are represented as mean ± SE. **(e)** CD163 co-localizes with CD68 indicating that CD163 is expressed by macrophages/microglia. DAPI is a nuclear stain.

**Figure 5:**
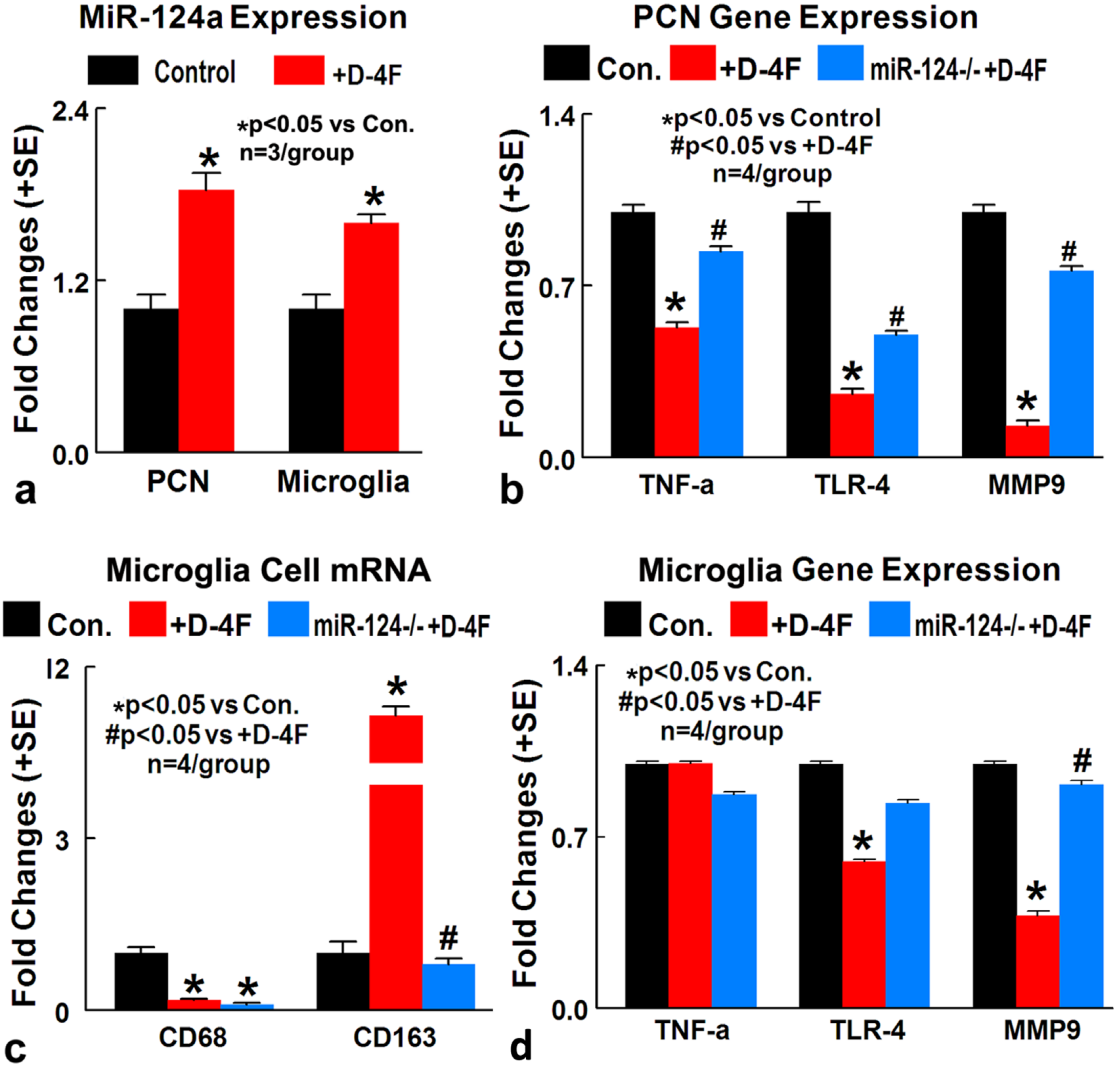
*In vitro*, D-4F decreases inflammatory factor expression in cultured PCN and promotes M2 macrophage polarization in cultured microglia cells under conditions of high glucose and OGD **(a)** D-4F treatment significantly increases miR-124a gene expression in cultured PCN and microglial cell under conditions of high glucose and OGD, compared to non-treatment control (^*^p<0.05, n=3/group). **(b)** MiR-124a inhibition in PCN cells significantly attenuates D-4F induced anti-inflammatory effects identified by significant increases in TNFα, MMP9 and TLR4 gene expression compared to D-4F treated PCN (^*^p<0.05 vs. control, ^#^p<0.05 vs. D-4F treatment, n=4/group). **(c)** MiR-124 knock down in microglia culture significantly attenuates D-4F induced M2-macrophage polarization (^*^p<0.05 vs. control, ^#^p<0.05 vs. D-4F treatment, n=4/group). **(d)** MiR-124a inhibition in microglial cells significantly attenuates D-4F induced anti-inflammatory effects identified by increases in MMP9 (^*^p<0.05 vs. control, ^#^p<0.05 vs. D-4F treatment, n=4/group) and TLR4 gene expression compared to D-4F treated microglia. Data are represented as mean ± SE.

To investigate mechanisms by which D-4F decreases BBB leakage and WM damage, we first test whether D-4F treatment of stroke decreases neuroinflammation after stroke in T1DM rats. TLR4, nuclear factor kappa-light-chain-enhancer of activated B cells (NFκB) and MMP9 expression were measured in the IBZ. D-4F treatment significantly (n=8/group, ^*^p<0.05) decreases TLR4 (Figure 3a), MMP9 (Figure 3b) and nuclear NFκB (Figure 3c) expression compared to PBS treated T1DM stroke control rats at 48 hours after stroke. D-4F treatment also significantly (n=8/group, ^*^p<0.05) promotes M2 macrophage polarization which is identified by increased CD163 expression (Figure 3d) compared to PBS treated T1DM stroke rats at 48 hours after stroke. Figure 5e shows that CD163 co-localizes with CD68 (expressed on monocytes/macrophages), indicating that CD163 is expressed by macrophages/microglia. Inflammatory factors were not detected in T1DM-sham control group.

**Figure 4:**
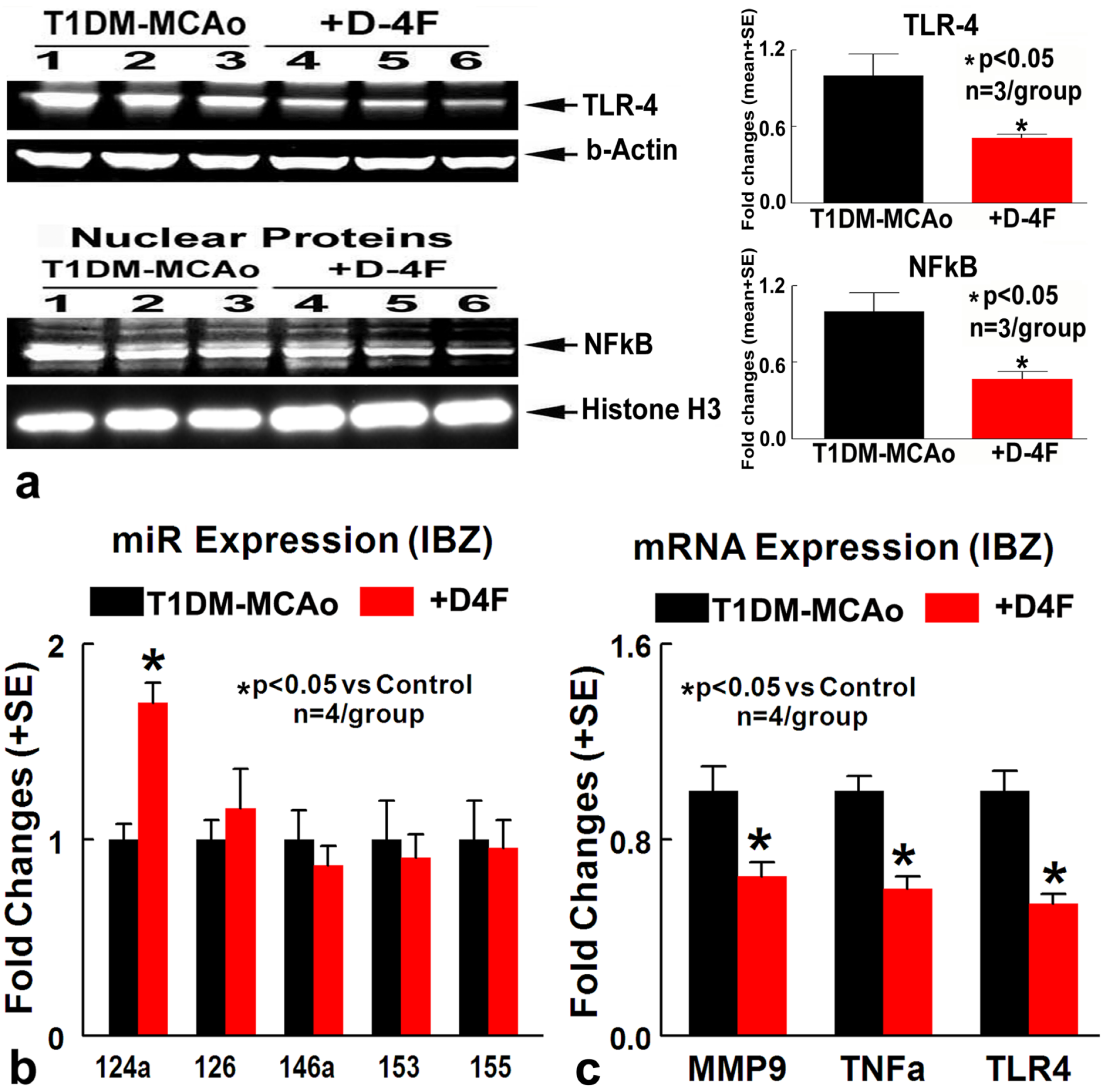
D-4F treatment of stroke in T1DM rats significantly decreases inflammatory factor expression and increases miR-124a expression **(a)** Western blot assay showing that D-4F treatment of T1DM stroke rats decreases TLR4, and nuclear NFκB expression in the ischemic brain compared to PBS treated T1DM stroke control rats (^*^p<0.05, n=3/group). **(b)** D-4F treatment significantly increases miR-124a expression in the ischemic brain at 48 hours after stroke (^*^p<0.05, n=4/group). **(c)** D-4F treatment significantly decreases MMP9, TLR4 and TNFα gene expression in the ischemic brain compared to PBS treated T1DM stroke control rats at 48 hours after stroke (^*^p<0.05, n=4/group). Data are represented as mean ± SE.

Consistent with immunostaining, Western blot assay show that D-4F treatment of T1DM stroke rats decreases TLR4, and nuclear NFκB expression in the ischemic brain (Figure 4a) compared to PBS treated T1DM stroke control rats. The data indicated that D-4F treatment significantly decreases inflammatory factor expression and increases M2 macrophage expression in the ischemic brain in T1DM stroke.

### D-4F treatment of stroke in T1DM rats significantly increases miR-124a expression and decreases inflammatory factor gene expression in the ischemic brain

MiRs can alter several genes, pathways, and biological networks as well as neuroinflammation [8]. To test whether the anti-inflammatory effects of D-4F are related to miR expression, expression of miRs (miR-124a, miR-126, miR-146a, miR-153, miR-155) related to inflammation, BBB integrity and DM were measured in the ischemic brain of control and D-4F treated T1DM stroke rats. Figure 4b shows that D-4F treatment significantly increases miR-124a expression in the ischemic brain at 48 hours after stroke (n=4/group, ^*^p<0.05). MiR-124 is known to modulate neuronal and immune processes [24]. MiR-124 decreases neuroinflammatory factor such as MMP9, TLR4 and TNFα [15, 25, 26]. Figure 4c shows that D-4F treatment significantly decreases MMP9, TLR4 and TNFα gene expression in the ischemic brain compared to PBS treated T1DM stroke rats (n=4/group, ^*^p<0.05). These data indicate that D-4F treatment of stroke in T1DM rats increases miR-124a expression, and decreases inflammatory factor gene expression in the ischemic brain.

### D-4F decreases inflammatory factor expression in cultured PCN and promotes M2 macrophage polarization in cultured microglia cells *in vitro*

MiR-124 is expressed in neurons in the developing and adult nervous systems, and promotes neurite outgrowth during neuronal differentiation [27]. MiR-124 is not only expressed in neurons, but is also expressed in microglia and is a key regulator of microglial quiescence in the CNS [11]. To further test whether D-4F treatment induced anti-inflammatory effects are derived from increased miR-124 activity, primary cortical neuron (PCN) and primary microglia cell cultures subjected to high glucose and oxygen glucose deprivation (OGD) conditions were employed. D-4F treatment significantly increases miR-124a gene expression in cultured PCN and microglial cells compared to non-treatment control (Figure 5a, ^*^p<0.05). In PCN cultures, D-4F treatment significantly decreases TNFα, MMP9 and TLR4 expression compared to non-treated control PCN. When miR-124a is inhibited in PCN cells, D-4F induced anti-inflammatory effects were significantly attenuated, as identified by significant increases in TNFα, MMP9 and TLR4 gene expression compared to D-4F treated PCN (Figure 5b, ^*^p<0.05).

In microglia culture, D-4F treatment significantly decreases MMP9 and TLR4 expression compared to non-treated control microglia. When miR-124a is inhibited in microglia cells, D-4F induced anti-inflammatory effects were significantly attenuated, as identified by increases in MMP9 gene expression compared to D-4F treated microglia (Figure 5d, p<0.05). D-4F treatment also significantly decreases M1 microglia and increases M2 macrophage polarization compared to non-treated microglia culture (^*^p<0.05). MiR-124 knock down in microglia culture, significantly attenuated D-4F induced M2-macrophage polarization (Figure 5c, ^*^p<0.05). These data indicate that miR-124 may at least partially contribute to D-4F induced anti-inflammatory effect and M2 macrophage polarization.

## DISCUSSION

In this study, for the first time to our knowledge, we demonstrate that D-4F treatment of T1DM rats subjected to stroke significantly increases miR-124a expression in the ischemic brain and in cultured PCN and microglial cells, decreases inflammatory factor expression, promotes M2 macrophage polarization, increases tight junction protein expression, decreases BBB leakage, decreases WM damage, and improves neurological functional outcome at 48 hours after stroke. The increase in miR-124a expression may partially facilitate the decrease of inflammatory responses and promote M2 macrophage polarization in T1DM stroke rats.

Increased BBB leakage and inflammatory responses in DM stroke rats are associated with worse functional outcome [3, 28]. In DM stroke patients, microglial activation is increased within minutes of ischemia onset and triggers the production of inflammatory cytokines, including MMP9 and TNFα, which exacerbate tissue damage [29]. The rapid activation of resident microglial cells is followed by the infiltration of blood-derived immune cells into the ischemic brain tissue within hours to a few days. The cerebrovascular endothelium is transformed from a quiescent state into a proinflammatory state by a cascade of molecular events that follow focal brain ischemia. Microglial cells and blood-derived monocytes/macrophages play key roles in inflammation during the onset as well as during the progressive aggravation of stroke lesions [30]. In this study, we found that D-4F treatment in T1DM rats subjected to stroke significantly decreases BBB leakage, increases tight junction protein expression, and reduces inflammatory factor expression such as MMP9, TNFα and NFκB in the ischemic brain, as well as improves functional outcome compared to PBS treated T1DM stroke rats at 48 hours after stroke. Therefore, control of BBB leakage and inflammatory responses may play an important role in D-4F induced improvement in functional outcome after stroke in T1DM rats.

The mechanism of D-4F induced decrease in BBB leakage is not clear. Tight junctions provide barriers between adjacent brain capillary endothelial cells at the BBB and stabilize blood vessels [31]. ZO-1 is a functionally critical tight junction protein [32]. In this study, we found that D-4F treatment significantly increases ZO-1 expression around blood vessels in the IBZ , which may contribute to the improved BBB integrity and function compared to PBS treated T1DM-stroke rats. DM-stroke significantly increases MMP9 activity compared to non-DM stroke, and MMP9 has been specifically implicated in the disruption of the BBB and neuronal cell death following cerebral ischemia [33, 34]. MMP9 is a neuroinflammatory factor that is elevated after stroke, and has been associated with WM injury, BBB disruption, worse neurological deficits and increased infarct volume after stroke [8, 35]. Diabetes induces an imbalance in the MMPs/TIMPs (tissue inhibitors of metalloproteinases) cascade and increases MMP9 which results in an increased degradation of occludin and collagen-IV, and subsequently increases BBB permeability facilitating increased infiltration of neutrophils into the infarct area [33]. In this study, we found that D-4F treatment significantly decreases MMP9 expression in the ischemic brain, which may contribute to the decreased BBB leakage.

BBB leakage and inflammation are closely related with WM damage after stroke [36, 37]. DM-stroke exacerbates BBB disruption and increases the expression of inflammatory factors including high mobility group box 1 (HMGB1), receptor for advanced glycation endproducts (RAGE), MMP9 and TLR4 in the ischemic brain, which have been correlated with increased WM damage in DM stroke animals [28, 34]. In this study, we found that D-4F treatment not only decreases BBB leakage and inflammatory factor expression, but also decreases WM damage identified by increased axon and myelin density in the ischemic boundary zone in T1DM rats. *in vitro* studies also indicate that D-4F decreases PCN inflammatory factor expression such as TNFα, MMP9 and TLR4.

Stroke induces inflammatory processes, characterized by rapid microglial activation, secretion of proinflammatory factors, and infiltration of inflammatory cells such as neutrophils, monocytes and macrophages into the ischemic brain tissue [38]. Abnormalities in innate immunity leading to diabetic complications have also been implicated in worse functional outcome after stroke [39, 40]. Macrophages constitute about 70% of infiltrating cells in the CNS inflammatory lesions [41]. “Classically activated” proinflammatory (M1) macrophages are the major inflammatory cells in the brain, and exert a neurotoxic function through the production of cytokines (Interleukins (IL)-1β and 6, TNFα) [42, 43]. “Alternatively activated” anti-inflammatory (M2) macrophages typically express Arginase I, mannose receptor (CD206) and CD163 [44–49], have a neurotrophic role and facilitate axon growth [50]. In this study, we found that D-4F treatment significantly increases M2 macrophage polarization compared to PBS treated T1DM stroke rats. *in vitro*, we also found that D-4F treatment significantly decreases inflammatory factor MMP9, TNFα and TLR4 expression as well as promotes M2 macrophage polarization in cultured microglia cells subjected to high glucose and hypoxic conditions. However, the mechanism of D-4F induced M2 macrophage polarization is not completely understood.

MiR-124is a critical promoter of multifaceted anti-inflammatory effects by inhibiting the production of proinflammatory cytokines such as IL6 and TNFα [27]. *In vitro*, miR-124 expression is down-regulated by activated microglia, and in the CNS, miR-124 levels correlate inversely with the state of microglial activation and macrophages [11]. MiR-124 also promotes neuron-microglia interaction and desensitizes neurons in an inflammatory environment [51]. Increasing miR-124by injection significantly increases neuronal survival and increases number of M2-like polarized microglia/macrophages [52], and suppresses experimental autoimmune encephalomyelitis symptoms and leukocyte infiltration in the CNS [11]. Increasing miR-124 expression in the brain of mice subjected to stroke via intravenous injection of miR-124 agomir significantly decreases infarct volume and attenuates neuronal cell death and late apoptosis [53]. Loss ofmiR-124decreases oligodendrocyte cell numbers and myelination of axonal projections in the ventral hindbrain [54]. In this study, we found that D-4F significantly increases miR-124a expression and decreases its target gene MMP9, TLR4 and TNFα, while promoting M2 macrophage polarization in the ischemic brain and in cultured PCN and microglia cells subjected to high glucose and OGD conditions. Inhibition of miR-124a expression in PCN attenuates D-4F induced anti-inflammatory effects. Inhibition of miR-124 in cultured microglia significantly decreases D-4F induced M2 macrophage polarization and attenuated the decreased MMP9 expression. Therefore, increase of miR-124a expression by D-4F treatment may promote M2 macrophage polarization and anti-inflammatory effects.

### Limitations

In this study, we show that D-4F promotes beneficial effects after stroke in T1DM rats, which may at least in part be attributed to up regulation of miR-124a. In this study, we found that D-4F decreases MMP9, TLR4 and TNFa inflammatory factor expression; however, inhibition of miR-124 only partially attenuates D-4F-induced decreasing MMP9 expression in cultured microglia. However, several other miR's may also act in concert to promote D-4F induced beneficial effects. Other miRs may also mediate D-4F induced anti-inflammatory effects. In addition, miR-124 regulates many target genes as well as inflammatory factors. MiR-124 directly or indirectly regulates MMP9, TNFa and TLR4 warranted investigated in future study. Causal mechanistic studies relating modulation of miR-124a by D-4F are warranted, as are investigations of long term effects of D-4F treatment in T1DM stroke.

### Conclusions

Treatment of stroke in T1DM rats with D-4F increases miR-124a expression, decreases neuroinflammatory responses and promotes M2 macrophage polarization which, in concert, may contribute towards decreasing BBB leakage and WM damage thereby improving functional outcome.

## MATERIALS AND METHODS

All experimental procedures were carried out in accordance with the NIH Guide for the Care and Use of Laboratory Animals and approved by the Institutional Animal Care and Use Committee of Henry Ford Hospital.

Adult Male Wistar rats (225-250 g, 3 months) were used to induce T1DM by a single intraperitoneal (i.p) injection of 60 mg/kg Streptozotocin (Sigma) [55]. Two weeks later, fasting blood glucose was tested using a glucose analyzer (Accu-Chek Compact System; Roche Diagnostics). Animals with fasting blood glucose >300 mg/dl were defined diabetic and included in the study.

### Embolic middle cerebral artery occlusion (MCAo) model and experimental groups

T1DM rats were anesthetized with 2% isoflurane in a jar for pre anesthetic, and spontaneously respired with 1.5% isoflurane in 2:1 N_2_O:O_2_ mixture using a facemask connected and regulated with a modified FLUOTEC 3 Vaporizer (Fraser Harlake). Rectal temperature was maintained at 37°C throughout the surgical procedure using a feedback regulated water heating system. Embolic MCAo was performed as previously described [56]. Rats were randomized and assigned to different groups (n=11/group) and were treated with 1) PBS (2 ml, i.p.); 2) D-4F (1 mg/kg, i.p.) at 2 hours, 24 hours and 48 hours after MCAo. The dose (1 mg/kg, i.p injection) of D-4F was selected according previous publication [21]. After a mortality of 30% in each group; n=8/group was employed for further analysis. Sham surgery was performed in the same way as the MCAo model without injecting the emboli (n=8/group).

### Neurological functional tests

At 2 hours, 24 hours and 48 hours after MCAo, an investigator who was blinded to the experimental groups performed mNSS evaluation, adhesive removal test and foot-fault test, as previously described. The testing procedure is briefly described below:

a) mNSS test [2, 57]: In the mNSS test, motor, sensory, balance and reflex actions are tested. The absence of a tested reflex or abnormal response is scored as one point and neurological function is scored between 0-18. An animal with a score of 0 indicates no neurological deficits, and an animal receiving a maximum score of 18 indicates maximum neurological deficits.

b) Foot-fault test [58]: This test evaluates sensorimotor function, motor coordination and limb placing deficits during locomotion [59]. The testing equipment consists of an elevated grid floor (45 cm × 30 cm), 2.5 cm higher than a solid base floor, with 2.5 cm × 2.5 cm diameter grid spacing. The animal is placed on the grid and allowed to move freely. During the animals movement on the wire grid using their paws, a fall or slip through a grid opening due to an inaccurate forelimb placement is recorded as a foot-fault. Data are presented as the percentage of foot-faults of the left paw over a 100 forelimb movements.

c) Adhesive removal test [57]: The adhesive removal test evaluates somatosensory deficits by using 2 small pieces of adhesive-backed paper dots (of equal size, 113.1 mm^2^) as bilateral tactile stimuli occupying the distal-radial region on the wrist of each forelimb. The time to remove each stimulus from forelimbs was recorded for 5 trials per day. Individual trials were separated by at least 5 minutes. All animals were familiarized with the testing environment. Prior to stroke surgery, all animals were trained for 3 consecutive days such that rats could remove the stickers within 10 seconds.

### Exclusion criteria

Rats were excluded from the study if they failed to remove the adhesive sticker within 10 seconds during the training sessions, i.e. exhibit pre-operative asymmetries [59]. Rats were also excluded if the mNSS score was less than 6 (possibly small to no lesion, condition improves regardless of treatment) or over 13 (very large lesion, poor survival, condition deteriorates regardless of treatment) at 2 hours after stroke.

### BBB leakage measurement

An additional set of T1DM-MCAo control rats and T1DM-MCAo+D-4F treatment rats (n=4/group) were prepared and sacrificed at 48 hours after MCAo. Evans-blue dye (2%) was injected intravenously 4 hours before sacrifice and fluorescence intensity was measured using a microplate fluorescence reader (excitation 620 nm and emission 680 nm) [60]. The amount of extravasated Evans blue dye was quantified as micrograms per ischemic hemisphere [60].

### Lesion volume and brain hemorrhage measurement

All brains were fixed by transcardial perfusion with 0.9% saline, followed by perfusion and immersion in 4% paraformaldehyde then embedded in paraffin. Seven coronal sections of tissue were processed and stained with hematoxylin and eosin (H&E) for calculation of lesion volume and presented as a percentage of the lesion compared with the contra-lateral hemisphere. Brain hemorrhage was measured by using H&E staining. The percentage areas of petechial and gross hemorrhage were measured in each histological section and summed.

### Histological assessments

A standard block was obtained from the center of the lesion (bregma −2 mm~+2 mm). A series of 6 μm thick sections was cut from the block. Every 10^th^ coronal section for a total 5 sections was used for immunohistochemical staining. Antibodies against ZO-1 (1:50, Zymed) NFκB (1:500; Abcam), TLR4 (1:100; Santa Cruz Biotechnology), MMP9 (1:500, Santa Cruz Biotechnology), CD163 (1:1000, Biorbyt) and CD68 (1:30, Serotec) were used. DAPI was used to stain nuclei. BS staining was used to stain axons and LFB was used to stain myelin as described previously [61]. Control experiments consisted of staining brain coronal tissue sections as outlined above, but non-immune serum was substituted for the primary antibody. The immunostaining analysis was performed by an investigator blinded to the experimental groups.

### Immunostaining quantification

Each slide containing 8 fields of view from the IBZ was digitized under a 40× or 20× objective (Olympus BX40) interfaced with an MCID image analysis system (Imaging Research). The IBZ is defined as the area surrounding the lesion. The data from five sections and eight regions within each section were averaged to obtain a single value for one animal and presented as percentage of positive area for TLR4, MMP9, BS and LFB, percentage number of nuclear positive cells for NFκB and positive cells number for CD163, respectively. For quantitation of ZO-1 expression, 10 large blood vessels in the IBZ were digitized under 40× and data presented as percentage of positive area.

### Primary cortical neuron culture

PCNs were harvested from pregnant (day 18) embryonic Wistar rats (Charles River). The cultures were prepared as previously described with some modifications [62, 63]. Briefly, the embryo cerebral cortex was dissected and dissociated in Ca^2+^ and Mg^2+^ free HBSS with 0.125% trypsin. The cells were plated on poly-d-lysine (Sigma) coated dishes (35 mm, Corning) and initially cultured in DMEM media (Life Technologies) with 5% fetal bovine serum for 5 hours, then neurobasal growth medium (Life Technologies) with 2% B-27 (Life Technologies), 2 mm GlutaMax, and 1% antibiotic-antimycotic was used.

### Primary microglia cell culture

Primary microglial cells were harvested following previously published methods [64]. Primary cortical tissue was harvested from pregnant Wistar day 18 rats (Charles River Laboratories). The cerebral cortex were dissected from the embryos and broken up in calcium and magnesium free HBSS with 0.125% trypsin for 10 minutes and then mechanically disassociated with a pipette (~20 strokes). The cells were filtered through a 40 μm cell strainer and plated at 5×10^6^ cells per T75 flask. The cells were cultured in 10 ml of growth media, DMEM media (Life Technologies) with 10% Fetal Bovine Serum (Life Technologies), with the media changed ever 2-3 days for ten days. On the tenth day the media was removed and 10ml of fresh growth media was added, the cap of the flask was wrapped in Parafilm to prevent atmosphere exchange, and the flask was shaken at 210 rpm for 1 hour on a Benchmark Incu-Shaker. After the shaking the media was collected and centrifuged to harvest the suspended microglia. Fresh growth media was added to the flask and the cells were allowed to culture for an additional week for a second shaking, to harvest more microglia. The populations obtained using this method were 90-95% pure for macrophage/microglia cell specific marker (Iba1). The harvested cells were then used for the studies, with no additional passages.

### MiR-124 knockdown in PCN and microglia using electroporation

To test effects of miR-124 in regulation of anti-inflammatory and M2 macrophage polarization, miR-124 inhibitor was employed to knockdown of PCN and microglia miR-124 expression. Briefly, a mixture of 100 μl Ingenio Electroporation Solution (Mirus) and 5 nmol rno-miR-124-3p inhibitor or mimic (GE Dharmacon) was prepared. Cells were harvested and 1-2×10^6^ were resuspended in the electroporation solution. The solution was loaded into Ingenio cuvettes with a 0.2 cm gap (Mirus) which were stored on ice until loaded into the Amaxa Nucleofection machine (Lonza) and electroporated using program Y-01. After electroporation cells were removed from the cuvettes and put into culture and allowed to grow for 48 hours before being used in the studies.

### Oxygen glucose deprivation (OGD)

To subject cells cultures to OGD, serum and glucose free media was used. Cells were placed in a hypoxia chamber (Forma Anaerobic System; Thermo Scientific) with 37°C incubator for 2 hours. After 2 hours, the cells were removed and replaced in high glucose cultured media (37.5 μM).

### Experimental treatment group *in vitro*

Cultured OGD-PCN and OGD-microglia were treated with: 1) non-treated control; 2) D-4F (50 ng/ml and 100 ng/ml); 3) miR-124-knockdown (miR-124-/-)+D-4F treatment. Gene expression using PCR and miR-124 expression was measured.

### Real time PCR

Total RNA was isolated with TRIzol (Invitrogen), then make cDNA using the M-MLV (Invitrogen) standard protocol. 2 μl cDNA was used to run a quantitative PCR using the SYBR Green real time PCR method using the following primers (Invitrogen).

TNFα: FWD: TACTCCCAGGTTCTCTTCAAGG;

REV: GGAGGTTGACTTTCTCCTGGTA;

MMP9: FWD: ATCTCTTCTAGAGACTGGGAAGGAG;

REV: AAGCTGATTGACTAAAGTAGCTGGA.

TLR4: FWD: TCTAACTTCCCTCCTGAGATGG

REV: ACTGGCTAGAGAGCAAGAGGAA

CD68: FWD: TGTTCAGCTCCAAGCCCAAA

REV: GTACCGTCACAACCTCCCTG

CD163: FWD: TGCTGTCACTAACGCTCCTG

REV: TCATTCATGCTCCAGCCGTT

The PCR was run in a ViiA 7 machine (Applied Biosystems) using a 3-stage program provided by the manufacturer, as follows; 2 minutes at 50°C, 10 minutes at 95°C, and then 40 cycles of 15 seconds at 95°C and 1 minute at 60°C. Glyceraldehyde-3-phosphate dehydrogenase (GAPDH) was used as the housekeeping gene for normalization. Each sample was tested in triplicate, and analysis of relative gene expression data using the 2^-ΔΔCT^ method.

### MiR measurement

Total RNA was isolated with TRIzol (Invitrogen), then make cDNA using the TaqMan miR Reverse transcription kit (Applied Biosystems) following standard protocol. 4 μl of cDNA it was used to run a TaqMan PCR following standard protocol, and using miR primers for miR-124a (Applied Biosystems).

### Western blot assay

Protein was isolated from samples using Trizol (Invitrogen). Protein concentration was measured using the BCA kit (Thermo Scientific) and 40 μg of protein/lane loaded in a 10% SDS PAGE precast gel (Invitrogen). Gel was transferred using the iBlot transfer system (Invitrogen). Nitrocellulose membrane was blocked in 2% I-Block (Applied Biosystems) in 1×TBS-T for one hour, and then either b-actin (Abcam, 1:10,000), Histone H3 (Cell Signaling, 1:1000), RAGE (R&D Biosystems, 1:500), TLR4 (Santa Cruz, 1:500), or NFκB (Abcam, 1:1,000) was used. Secondary antibodies (anti-mouse, Jackson ImmunoResearch) were added at 1:1,000 dilution in 2% I-Block in 1×TBS-T on a room temperature. The membranes were washed with 1×TBS-T, and then Luminol Reagent (Santa Cruz) was added. The membranes were then developed using a FluorChem E Imager system (ProteinSimple).

### Statistical analysis

One-way Analysis of Variance (ANOVA) was used for the evaluation of functional outcome and histology, respectively. “Contrast/estimate” statement was used to test the group difference. If an overall treatment group effect was detected at p<0.05, pair-wise comparisons were made. All data are presented as mean ± standard error (SE).

## Abbreviations

ANOVA: One-way Analysis of Variance
ApoA-1: Apolipoprotein A-1
BBB: Blood brain barrier
BS: Bielschowsky silver
CNS: Central nervous system
DM: Diabetes mellitus
FWD: Forward
GAPDH: Glyceraldehyde-3-phosphate dehydrogenase
H&E: Hematoxylin and eosin
HDL: High-density lipoprotein
HMGB1: High mobility group box 1
IBZ: Ischemic border zone
IL: Interleukin
LFB: Luxol fast blue
MCAo: Middle Cerebral Artery Occlusion
MiR: MicroRNA
MMP9: Matrix metalloproteinase 9
mNSS: Modified neurological severity score
NFκB: Nuclear factor kappa-light-chain-enhancer of activated B cells
OGD: Oxygen glucose deprivation
PBS: Phosphate buffered saline
PCN: Primary cortical neuron
PCR: Polymerase chain reaction
RAGE: Receptor for advanced glycation endproducts
REV: Reverse
SE: Standard error
T1DM: Type one DM
TIMPs: Tissue inhibitors of metalloproteinases
TLR4: Toll-like receptor-4
TNFα: Tumor necrosis factor-α
tPA: Tissue plasminogen activator
WM: White matter
ZO-1: Zona occluden-1
